# Producing the appropriate model and drug for intratumoural ablation

**DOI:** 10.1038/s41416-020-0895-6

**Published:** 2020-05-27

**Authors:** Christoforos Kosmidis, Chrysanthi Sardeli, Paul Zarogoulidis, Wolfgang Hohenforst-Schmidt, Anastasios Vagionas, Nikolaos Ioannis-Katsios, Konstantinos Sapalidis

**Affiliations:** 1 3rd University General Department, “AHEPA“ University Hospital, Aristotle University of Thessaloniki, Thessaloniki, Greece; 2 Department of Pharmacology & Clinical Pharmacology, School of Medicine, Faculty of Health Sciences, Aristotle University of Thessaloniki, Thessaloniki, Greece; 3 Sana Clinic Group Franken, Department of Cardiology/Pulmonology/Intensive Care/Nephrology, ‘Hof’ Clinics, University of Erlangen, Hof, Germany; 4 Oncology Department, General Hospital of Kavala, Kavala, Greece

**Keywords:** Cancer therapy, Drug delivery

## Abstract

Local treatment is necessary for several cancer patients. There are situations where cancer tissue induces locally severe symptoms. Therefore, additional local disease control is necessary. There are two major issues for efficient local treatment: the method of application and the penetration of the drug formulation. We need efficient tools to guide the drug formulation to the target point, and an effective drug formulation that is diffused within the tumour microenvironment with a sustained-release effect.

## Main

There are several types of cancer and each one has its own therapies. However, there are situations where local treatment modalities are necessary and therefore, we should have an efficient delivery system along with efficient drug formulations. Currently, there are ablation systems with radiofrequency, microwave and thermosphere.^1,2^ Moreover, these systems have been used along with drugs and gene therapy in order to enhance their antenna treatment effect.^3,4^ The application of the antenna can be done transthoracically, under CT guidance, inside an open surgery under ultrasound guidance, or even endoscopically.^1,2,5^ Several experiments have been performed initially in animal models, in order to evaluate the tumour necrosis effect and drug absorption in pathology findings.^4^ In the case that we choose to apply a drug for local treatment after choosing the right application system, we have to evaluate the diffusion of the drug within the tumour. Specifically, the tumour microenvironment plays a crucial role for the local drug absorption.^6^ Tumours with a high strain ratio have decreased permeability since their matrix is very thick/stiff. This information is provided with elastography.^7,8^ The drug formulation that we want to apply must have several characteristics in order to be efficient. Firstly, it should have a sustained-release local effect, and secondly, it should have a rheology that allows increased diffusion. Several groups have tried different drug formulations as local treatment. In the study by Gaballa et al.,^9^ local administration with ethanol-free paclitaxel was administered for insulinomas with the help of endoscopical ultrasound (EUS). Before this experiment, the drug paclitaxel was administered with ethanol; however, several undesirable inflammatory side effects on local tissue and vessels were observed from this drug formulation. Indeed, local disease control was observed, and the drug formulation remained effective even without the ethanol. In the study by Racioppi et al.,^10^ mitomycin C (MMC) was used as follows: intravesical MMC three times per week for 2 weeks for group 1. Transurethral resection of bladder tumour (TUR-BT) and early instillation and a weekly schedule of intravesical MMC for group 2. The authors wanted to investigate the best treatment approach/dosage/schedule. In the study by Hohenforst-Schmidt et al.,^4^ the authors investigated whether the drug lipiodol with or without cisplatin and microwave ablation enhances the treatment effect of microwave and platinum analogues in an animal model. Unfortunately, lipiodol did not diffuse efficiently within the tumour and in the magnetic resonance (MRI) that was performed in every test subject; concentrations of the drug lipiodol could be observed within the injection site. The addition of the drug lipiodol did not have any additional effect as a combination therapy with microwave ablation and/or cisplatin. In the study by Hohenforst-Schmidt et al.,^3^ a novel (at that time) non-viral vector 2-diethylaminoethyl-dextran methyl methacrylate (DDMC) was combined with p53 and administered locally with or without microwave ablation and cisplatin as a combination treatment. Again, on pathology specimens from the tumour after injection of the DDMC–p53 complex, thick concentrations could be observed in the injection sites. The DDMC–p53 complex was too thick to be efficiently diffused within the tumour. The treatment had higher efficiency when cisplatin/microwave and DDMC–p53 were combined. In the study by Duffy et al.,^11^ patients with hepatocellular carcinoma (HCC) were enrolled, and radiofrequency ablation (RF) with or without tremelimumab (immunotherapy) was applied. This study was based on the concept that cancer cell apoptosis by direct methods (known as ablation) can result in the immune system being activated (or switched on). The immune system could easily then recognise and target the cancer cells in the local tissue. Moreover, drugs available known as immune checkpoint inhibitors (such as tremelimumab) could enhance this effect. Indeed, the combination of local RF with immunotherapy had a combo effect and higher treatment efficiency. In the study by Rogers et al.,^12^ local chemoablation was performed under real-time MRI. This could be a method to observe the diffusion of the drug formulation within tumours. In the study by Xu et al.,^13^ post-radiotherapy oesophageal cancer patients with mediastinal lymph node recurrence were enrolled in this study. Patients were randomised into the radiation (±chemotherapy) or the chemoablation group. Patients randomised to the chemoradiotherapy group received additional radiotherapy, second-line chemotherapy or both. Patients randomised to the chemoablation group received CT-guided percutaneous chemical ablation. Clinical remission was assessed at 1 month by contrast CT. The drug formulation was administered on the tumour site, and as observed by the re-evaluation of the authors, was efficiently used. In the review paper by Rehman et al.,^14^ a wide search since 1965–2003 was performed regarding tissue chemoablation. The review reviewed articles regarding both in vitro and in vivo experiments, and the data included were regarding the following: 95% ethanol (4 ml), 24% hypertonic saline (4 ml) or 50% acetic acid (4 ml) solutions as well as in gel form. The conclusions were as follows: to date, ethanol chemoablation has been shown to be feasible and reproducible only for metastatic hepatic carcinoma. On the other hand, in urology, chemoablation is still very much in the investigational stage for both the prostate and the kidney cancer. A significant drawback is that even in the gel form, the spread of the chemoablative substance through the tissue is irregular and unpredictable. In the future, chemoablation may become a more effective modality by combining it with radiofrequency or other energy sources. Local chemoablation techniques have changed from 1965 to 2020. We have new methods to evaluate the diffusion of a drug within the tumour, and also to guide the drug in the proper site of administration. Gels due to their rheology are more difficult to handle and control their diffusion within a tumour. In the study by Rossi et al.,^15^ in this issue of the *British Journal of Cancer*, the authors created a poloxamer-based thermoresponsive hydrogel, suitable for intratumoural administration and retention. This drug formulation demonstrated preliminary evidence of local tumour control, both in vivo and in vitro with minimal off-site toxicity. In order to complete local tumour control, we need a combination of sustained-release drug, visualisation during application within the tumour and of course the appropriate ‘main drug' for each cancer type. Poloxamer-based thermoresponsive hydrogels could certainly be the basic solution for treatment combinations. Ablation systems, such as RF, microwave and thermosphere, can be used for additional treatment effects. Thermosphere ablation antennas have the advantage of a homogeneous therapeutic effect indifferent of the tissue strain ratio.

## Data Availability

Not applicable.
